# 3,3′-[1,2-Phenyl­enebis(methyl­ene)]bis­(1-ethyl-1*H*-benzimidazol-1-ium) bis­(hexa­flourophosphate)

**DOI:** 10.1107/S1600536812019344

**Published:** 2012-05-05

**Authors:** Rosenani A. Haque, Muhammad Adnan Iqbal, Mohd Mustaqim Rosli, Hoong-Kun Fun

**Affiliations:** a School of Chemical Sciences, Universiti Sains Malaysia, 11800 USM, Penang, Malaysia; b X-ray Crystallography Unit, School of Physics, Universiti Sains Malaysia, 11800 USM, Penang, Malaysia

## Abstract

In the title compound, C_26_H_28_N_4_^2+^·2PF_6_^−^, the complete cation is generated by a crystallographic twofold axis. The benz­imidazole ring is almost planar (r.m.s. deviation = 0.0207 Å) and makes dihedral angles of 50.12 (2)° with its symmetry-related component and 65.81 (2)° with the central benzene ring. In the crystal, mol­ecules are linked into a three-dimensional network by C—H⋯F inter­actions. A π–π inter­action with a centroid–centroid distance of 3.530 (1) Å is observed. Four F atoms of the hexa­fluoro­phosphate anion are disordered over two sets of sites in a 0.889 (6):0.111 (6) ratio.

## Related literature

For the biological applications of benzimidazoles, see: Narasimhan *et al.* (2012 ▶). For a related structure, see: Haque *et al.* (2012 ▶).
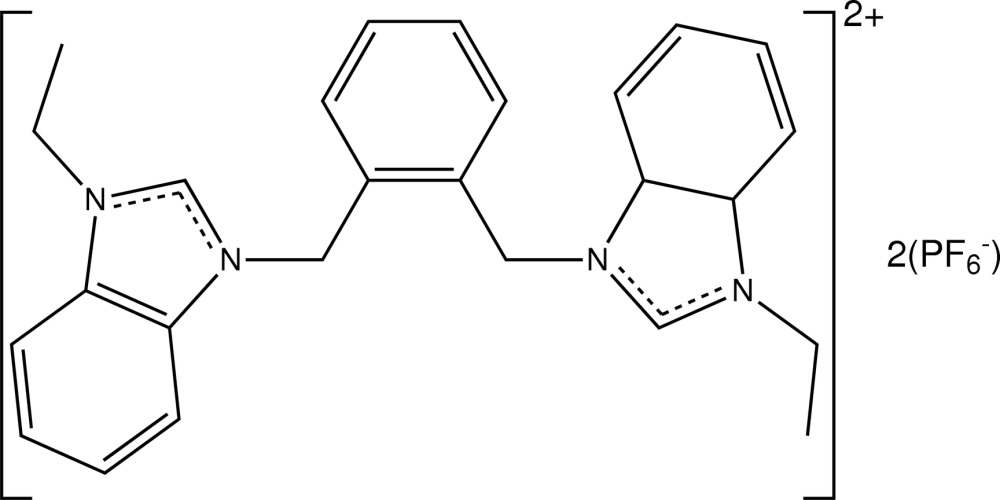


## Experimental

### 

#### Crystal data

C_26_H_28_N_4_^2+^·2PF_6_^−^
*M* *_r_* = 686.46
Monoclinic, 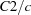

*a* = 23.2016 (5) Å
*b* = 8.1526 (2) Å
*c* = 16.9992 (4) Å
β = 121.274 (1)°
*V* = 2748.23 (11) Å^3^
*Z* = 4
Mo *K*α radiation
μ = 0.27 mm^−1^
*T* = 100 K
0.26 × 0.26 × 0.14 mm

#### Data collection

Bruker SMART APEXII CCD diffractometer
Absorption correction: multi-scan (*SADABS*; Bruker, 2009 ▶) *T*_min_ = 0.933, *T*_max_ = 0.963
14787 measured reflections
3921 independent reflections
3156 reflections with *I* > 2σ(*I*)
*R*_int_ = 0.037

#### Refinement

*R*[*F* ^2^ > 2σ(*F* ^2^)] = 0.046
*wR*(*F* ^2^) = 0.098
*S* = 1.05
3921 reflections
217 parameters
H-atom parameters constrained
Δρ_max_ = 0.48 e Å^−3^
Δρ_min_ = −0.35 e Å^−3^

### 

Data collection: *APEX2* (Bruker, 2009 ▶); cell refinement: *SAINT* (Bruker, 2009 ▶); data reduction: *SAINT*; program(s) used to solve structure: *SHELXTL* (Sheldrick, 2008 ▶); program(s) used to refine structure: *SHELXTL*; molecular graphics: *SHELXTL*; software used to prepare material for publication: *SHELXTL* and *PLATON* (Spek, 2009 ▶).

## Supplementary Material

Crystal structure: contains datablock(s) I, global. DOI: 10.1107/S1600536812019344/hb6760sup1.cif

Structure factors: contains datablock(s) I. DOI: 10.1107/S1600536812019344/hb6760Isup2.hkl

Additional supplementary materials:  crystallographic information; 3D view; checkCIF report

## Figures and Tables

**Table 1:** Hydrogen-bond geometry (Å, °)

*D*—H⋯*A*	*D*—H	H⋯*A*	*D*⋯*A*	*D*—H⋯*A*
C1—H1*A*⋯F6^i^	0.95	2.42	3.142 (3)	133
C3—H3*A*⋯F5^ii^	0.95	2.45	3.374 (2)	166
C5—H5*A*⋯F5^iii^	0.95	2.52	3.420 (3)	159
C6—H6*A*⋯F4^iv^	0.95	2.53	3.392 (2)	151
C8—H8*B*⋯F1^i^	0.99	2.39	3.350 (3)	164
C13—H13*C*⋯F1^v^	0.98	2.55	3.523 (2)	174
C13—H13*C*⋯F5^v^	0.98	2.50	3.166 (2)	125

Symmetry codes: (i) 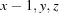; (ii) 
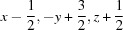
; (iii) 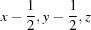; (iv) 
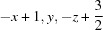
; (v) 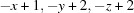.

**Fig. 1.**
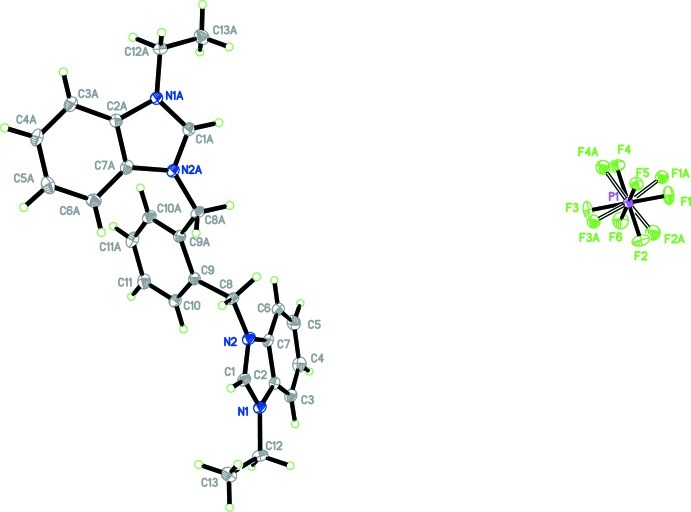
The molecular structure, showing 50% probability displacement ellipsoids. Hydrogen atoms are shown as spheres of arbitrary radius.

**Fig. 2.**
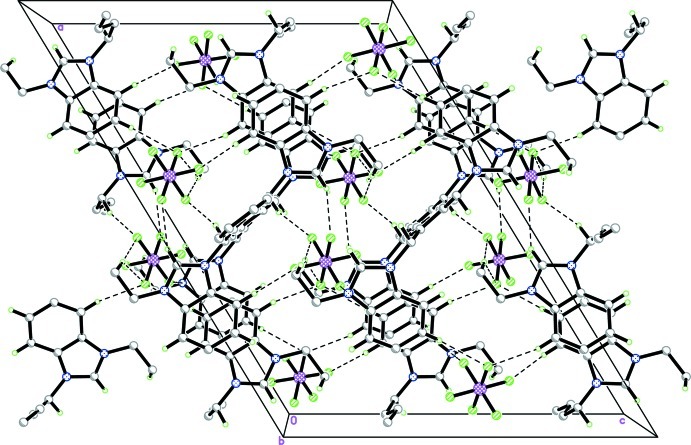
The crystal packing of (I). Dashed lines indicate hydrogen bonds. H atoms not involved in the hydrogen bond interactions have been omitted for clarity.

## supplementary crystallographic information

### Comment

Benzimidazole-constituted compounds are known as bioactive heterocyclic compounds that are of wide interest for biological and clinical applications (Narasimhan *et al.*, 2012). As a part of our ongoing studies in this area (Haque *et al.*, 2012), we now describe the title compound.

All parameters in the title compound (Fig. 1) are within normal ranges. The complete molecule is generated by a crystallographic two-fold axis. The benzimidazole (N1—N2/C1—C7) ring is planar with the r.m.s 0.0207 Å. It makes a dihedral angle of 50.12 (2)° with its symmetrical component and 65.81 (2) Å with the central benzene ring (C9—C11/C9A—C11A). Four fluorine atoms (F1—F4) of the hexafluorophosphate cation are disordered over two positions with the final refined occupancies of 0.889 (6):0.111 (6).

In the crystal structure, (Fig. 2), the molecules are linked into a three-dimensional network through intermolecular C—H···F hydrogen bonds (Table 1). A π-π interaction with centroid-centroid distance of 3.530 (1) Å is observed (*Cg*1 = C2—C7).

### Experimental

A mixture of benzimidazole (1.18 g, 10 mmol) and potassium hydroxide (1.18 g, 15 mmol) in 30 ml of DMSO was stirred at room temperature (27–28°C) for 30 min. Ethyl bromide (0.75 ml, 10 mmol) was added drop-wise into this consistently stirring mixture and it was further stirred for 2 h at same temperature, then poured into water (150 ml) and was extracted by chloroform (3 × 20 ml). The extract was filtered by five plies of filter papers with medium porosity to collect a crystal-clear solution which was evaporated under vacuum to get *N*-ethylbenzimidazole (I) as a thick yellowish fluid. Then, a mixture of I (0.73 g, 5 mmol) and 1,2- bis(bromomethyl)benzene (0.66 g, 2.5 mmol) in 1,4-dioxane (20 ml) was refluxed at 110°C for 12 h. The bromide salt of title compound appeared as beige-colored precipitates in a dark brown solution. The mixture was filtered and the precipitates were washed by fresh 1,4-dioxane (3 *x* 5 ml) and dried at room temperature for 24 h. The soft lumps so obtained were dissolved in methanol (30 ml) along with KPF_6_ (1.84 g, 10 mmol) and stirred for 3 h at room temperature. The white solid that appeared was filtered, washed by fresh methanol followed by water. The compound was dried at room temperature (1.53 g, 89.5%). A saturated solution of 2.2PF_6_ dissolved in acetonitrile (1 ml) was prepared. The compound was dissolved in it and the solution was evaporated at room temperature to collect colourless blocks of the title compound.

### Refinement

All H atoms attached to C atoms were fixed geometrically and refined as riding with C—H = 0.95–0.99 Å and *U*_iso_(H) = 1.2*U*_eq_(C) or 1.5*U*_eq_(*C*-methyl). A rotating group model was applied to the methyl group. Four fluorine atoms (F1—F4) of the hexafluorophosphate cation are disordered over two positions with the final refined occupancies of 0.889 (6):0.111 (6).The minor component was refined isotropically.

### Crystal data

**Table:**

C_26_H_28_N_4_^2+^·2PF_6_^−^	*F*(000) = 1400
*M**_r_* = 686.46	*D*_x_ = 1.659 Mg m^−^^3^
Monoclinic, *C*2/*c*	Mo *K*α radiation, λ = 0.71073 Å
Hall symbol: -C 2yc	Cell parameters from 3899 reflections
*a* = 23.2016 (5) Å	θ = 2.5–29.7°
*b* = 8.1526 (2) Å	µ = 0.27 mm^−^^1^
*c* = 16.9992 (4) Å	*T* = 100 K
β = 121.274 (1)°	Block, colourless
*V* = 2748.23 (11) Å^3^	0.26 × 0.26 × 0.14 mm
*Z* = 4	

### Data collection

**Table:**

Bruker SMART APEXII CCD diffractometer	3921 independent reflections
Radiation source: fine-focus sealed tube	3156 reflections with *I* > 2σ(*I*)
Graphite monochromator	*R*_int_ = 0.037
φ and ω scans	θ_max_ = 29.8°, θ_min_ = 2.1°
Absorption correction: multi-scan (*SADABS*; Bruker, 2009)	*h* = −32→31
*T*_min_ = 0.933, *T*_max_ = 0.963	*k* = −11→9
14787 measured reflections	*l* = −23→23

### Refinement

**Table:**

Refinement on *F*^2^	Primary atom site location: structure-invariant direct methods
Least-squares matrix: full	Secondary atom site location: difference Fourier map
*R*[*F*^2^ > 2σ(*F*^2^)] = 0.046	Hydrogen site location: inferred from neighbouring sites
*wR*(*F*^2^) = 0.098	H-atom parameters constrained
*S* = 1.05	*w* = 1/[σ^2^(*F*_o_^2^) + (0.0339*P*)^2^ + 4.2095*P*] where *P* = (*F*_o_^2^ + 2*F*_c_^2^)/3
3921 reflections	(Δ/σ)_max_ = 0.001
217 parameters	Δρ_max_ = 0.48 e Å^−^^3^
0 restraints	Δρ_min_ = −0.35 e Å^−^^3^

### Special details

**Table:**

Experimental. The crystal was placed in the cold stream of an Oxford Cryosystems Cobra open-flow nitrogen cryostat (Cosier & Glazer, 1986) operating at 100.0 (1) K.
Geometry. All e.s.d.'s (except the e.s.d. in the dihedral angle between two l.s. planes) are estimated using the full covariance matrix. The cell e.s.d.'s are taken into account individually in the estimation of e.s.d.'s in distances, angles and torsion angles; correlations between e.s.d.'s in cell parameters are only used when they are defined by crystal symmetry. An approximate (isotropic) treatment of cell e.s.d.'s is used for estimating e.s.d.'s involving l.s. planes.
Refinement. Refinement of *F*^2^ against ALL reflections. The weighted *R*-factor *wR* and goodness of fit *S* are based on *F*^2^, conventional *R*-factors *R* are based on *F*, with *F* set to zero for negative *F*^2^. The threshold expression of *F*^2^ > σ(*F*^2^) is used only for calculating *R*-factors(gt) *etc*. and is not relevant to the choice of reflections for refinement. *R*-factors based on *F*^2^ are statistically about twice as large as those based on *F*, and *R*- factors based on ALL data will be even larger.

### Fractional atomic coordinates and isotropic or equivalent isotropic displacement parameters (Å^2^)

**Table:**

	*x*	*y*	*z*	*U*_iso_*/*U*_eq_	Occ. (<1)
P1	0.89811 (2)	0.80829 (6)	0.89390 (3)	0.01579 (11)	
F5	0.84112 (5)	0.94532 (13)	0.83897 (7)	0.0220 (2)	
F6	0.95467 (6)	0.67136 (15)	0.94831 (8)	0.0323 (3)	
F1	0.95400 (8)	0.92423 (17)	0.89472 (17)	0.0309 (5)	0.889 (6)
F2	0.91321 (10)	0.8889 (2)	0.98909 (10)	0.0303 (5)	0.889 (6)
F3	0.84260 (11)	0.6929 (2)	0.8940 (2)	0.0311 (5)	0.889 (6)
F4	0.88302 (12)	0.7290 (2)	0.79927 (10)	0.0314 (5)	0.889 (6)
F1A	0.9350 (7)	0.9043 (15)	0.8457 (11)	0.024 (3)*	0.111 (6)
F2A	0.9323 (8)	0.9133 (18)	0.9770 (10)	0.032 (4)*	0.111 (6)
F4A	0.8573 (8)	0.7032 (18)	0.7950 (10)	0.029 (3)*	0.111 (6)
F3A	0.8547 (9)	0.713 (2)	0.9237 (11)	0.024 (4)*	0.111 (6)
N1	0.16728 (7)	0.66335 (17)	1.09095 (9)	0.0146 (3)	
N2	0.10920 (7)	0.67626 (18)	0.94040 (9)	0.0154 (3)	
C1	0.10867 (8)	0.7060 (2)	1.01737 (11)	0.0157 (3)	
H1A	0.0717	0.7513	1.0192	0.019*	
C2	0.20844 (8)	0.5997 (2)	1.06084 (11)	0.0142 (3)	
C3	0.27289 (8)	0.5312 (2)	1.10913 (12)	0.0174 (3)	
H3A	0.2982	0.5254	1.1744	0.021*	
C4	0.29766 (9)	0.4723 (2)	1.05606 (13)	0.0206 (4)	
H4A	0.3410	0.4226	1.0859	0.025*	
C5	0.26085 (9)	0.4833 (2)	0.95923 (13)	0.0212 (4)	
H5A	0.2802	0.4424	0.9257	0.025*	
C6	0.19723 (9)	0.5525 (2)	0.91211 (12)	0.0174 (3)	
H6A	0.1723	0.5610	0.8469	0.021*	
C7	0.17168 (8)	0.6091 (2)	0.96517 (11)	0.0150 (3)	
C8	0.05184 (8)	0.6967 (2)	0.84619 (11)	0.0163 (3)	
H8A	0.0663	0.7624	0.8105	0.020*	
H8B	0.0157	0.7582	0.8480	0.020*	
C9	0.02370 (8)	0.5336 (2)	0.79767 (11)	0.0151 (3)	
C10	0.04406 (8)	0.3851 (2)	0.84445 (12)	0.0178 (3)	
H10A	0.0740	0.3845	0.9093	0.021*	
C11	0.02104 (9)	0.2372 (2)	0.79734 (12)	0.0200 (4)	
H11A	0.0342	0.1364	0.8301	0.024*	
C12	0.18631 (9)	0.6741 (2)	1.18835 (11)	0.0186 (3)	
H12A	0.2176	0.7675	1.2179	0.022*	
H12B	0.2104	0.5727	1.2208	0.022*	
C13	0.12542 (9)	0.6968 (2)	1.19816 (13)	0.0212 (4)	
H13A	0.1402	0.7015	1.2636	0.032*	
H13B	0.0944	0.6044	1.1692	0.032*	
H13C	0.1024	0.7992	1.1681	0.032*	

### Atomic displacement parameters (Å^2^)

**Table:**

	*U*^11^	*U*^22^	*U*^33^	*U*^12^	*U*^13^	*U*^23^
P1	0.0129 (2)	0.0162 (2)	0.0174 (2)	0.00054 (16)	0.00725 (17)	0.00099 (17)
F5	0.0197 (5)	0.0208 (5)	0.0221 (5)	0.0054 (4)	0.0084 (4)	0.0053 (4)
F6	0.0233 (6)	0.0279 (6)	0.0390 (7)	0.0116 (5)	0.0115 (5)	0.0080 (5)
F1	0.0212 (8)	0.0261 (7)	0.0505 (14)	−0.0047 (6)	0.0224 (9)	−0.0001 (7)
F2	0.0303 (9)	0.0381 (9)	0.0169 (6)	0.0070 (7)	0.0083 (6)	−0.0039 (6)
F3	0.0263 (9)	0.0207 (8)	0.0519 (15)	−0.0042 (7)	0.0244 (11)	0.0045 (10)
F4	0.0382 (11)	0.0331 (9)	0.0228 (7)	0.0058 (8)	0.0157 (7)	−0.0062 (6)
N1	0.0127 (6)	0.0161 (7)	0.0134 (6)	0.0006 (5)	0.0056 (5)	−0.0010 (5)
N2	0.0112 (6)	0.0185 (7)	0.0137 (6)	0.0007 (5)	0.0044 (5)	−0.0010 (6)
C1	0.0122 (7)	0.0179 (8)	0.0151 (7)	0.0008 (6)	0.0057 (6)	−0.0011 (6)
C2	0.0126 (7)	0.0133 (7)	0.0157 (7)	−0.0015 (6)	0.0067 (6)	−0.0014 (6)
C3	0.0132 (8)	0.0175 (8)	0.0175 (8)	0.0002 (6)	0.0051 (7)	0.0007 (7)
C4	0.0131 (8)	0.0216 (9)	0.0238 (9)	0.0034 (7)	0.0074 (7)	0.0021 (7)
C5	0.0198 (9)	0.0219 (9)	0.0268 (9)	0.0000 (7)	0.0155 (8)	−0.0023 (8)
C6	0.0168 (8)	0.0189 (8)	0.0161 (8)	−0.0025 (6)	0.0082 (7)	−0.0023 (7)
C7	0.0112 (7)	0.0142 (8)	0.0165 (8)	−0.0017 (6)	0.0050 (6)	−0.0009 (6)
C8	0.0117 (7)	0.0202 (8)	0.0114 (7)	−0.0004 (6)	0.0022 (6)	0.0006 (6)
C9	0.0102 (7)	0.0201 (8)	0.0143 (8)	−0.0008 (6)	0.0059 (6)	−0.0009 (6)
C10	0.0117 (8)	0.0235 (9)	0.0147 (8)	−0.0010 (6)	0.0045 (6)	0.0018 (7)
C11	0.0146 (8)	0.0191 (8)	0.0227 (9)	0.0014 (6)	0.0072 (7)	0.0042 (7)
C12	0.0193 (8)	0.0216 (9)	0.0121 (7)	0.0021 (7)	0.0062 (7)	−0.0011 (7)
C13	0.0260 (9)	0.0195 (9)	0.0227 (9)	0.0017 (7)	0.0158 (8)	0.0017 (7)

### Geometric parameters (Å, º)

**Table:**

P1—F2A	1.481 (14)	C4—C5	1.410 (3)
P1—F3A	1.552 (19)	C4—H4A	0.9500
P1—F4	1.5947 (14)	C5—C6	1.382 (2)
P1—F3	1.5957 (18)	C5—H5A	0.9500
P1—F1	1.5987 (13)	C6—C7	1.390 (2)
P1—F6	1.6011 (12)	C6—H6A	0.9500
P1—F2	1.6077 (15)	C8—C9	1.522 (2)
P1—F5	1.6082 (11)	C8—H8A	0.9900
P1—F1A	1.656 (11)	C8—H8B	0.9900
P1—F4A	1.674 (14)	C9—C10	1.390 (2)
N1—C1	1.330 (2)	C9—C9^i^	1.409 (3)
N1—C2	1.397 (2)	C10—C11	1.391 (3)
N1—C12	1.478 (2)	C10—H10A	0.9500
N2—C1	1.337 (2)	C11—C11^i^	1.383 (3)
N2—C7	1.395 (2)	C11—H11A	0.9500
N2—C8	1.467 (2)	C12—C13	1.518 (2)
C1—H1A	0.9500	C12—H12A	0.9900
C2—C7	1.392 (2)	C12—H12B	0.9900
C2—C3	1.395 (2)	C13—H13A	0.9800
C3—C4	1.383 (2)	C13—H13B	0.9800
C3—H3A	0.9500	C13—H13C	0.9800
			
F2A—P1—F3A	95.5 (8)	N1—C1—H1A	124.8
F2A—P1—F4	157.6 (7)	N2—C1—H1A	124.8
F3A—P1—F4	106.7 (6)	C7—C2—C3	121.92 (15)
F2A—P1—F3	111.9 (7)	C7—C2—N1	106.67 (14)
F4—P1—F3	90.44 (11)	C3—C2—N1	131.37 (15)
F2A—P1—F1	67.6 (7)	C4—C3—C2	115.88 (16)
F3A—P1—F1	162.7 (6)	C4—C3—H3A	122.1
F4—P1—F1	90.05 (9)	C2—C3—H3A	122.1
F3—P1—F1	179.50 (11)	C3—C4—C5	122.29 (16)
F2A—P1—F6	88.8 (6)	C3—C4—H4A	118.9
F3A—P1—F6	86.5 (7)	C5—C4—H4A	118.9
F4—P1—F6	89.14 (8)	C6—C5—C4	121.35 (16)
F3—P1—F6	90.47 (9)	C6—C5—H5A	119.3
F1—P1—F6	89.39 (7)	C4—C5—H5A	119.3
F3A—P1—F2	73.4 (6)	C5—C6—C7	116.48 (16)
F4—P1—F2	179.76 (10)	C5—C6—H6A	121.8
F3—P1—F2	89.66 (11)	C7—C6—H6A	121.8
F1—P1—F2	89.86 (9)	C6—C7—C2	122.06 (15)
F6—P1—F2	91.07 (7)	C6—C7—N2	131.37 (16)
F2A—P1—F5	91.4 (6)	C2—C7—N2	106.53 (14)
F3A—P1—F5	93.4 (7)	N2—C8—C9	112.54 (14)
F4—P1—F5	90.69 (7)	N2—C8—H8A	109.1
F3—P1—F5	89.39 (9)	C9—C8—H8A	109.1
F1—P1—F5	90.75 (7)	N2—C8—H8B	109.1
F6—P1—F5	179.78 (9)	C9—C8—H8B	109.1
F2—P1—F5	89.10 (7)	H8A—C8—H8B	107.8
F2A—P1—F1A	92.3 (7)	C10—C9—C9^i^	119.25 (10)
F3A—P1—F1A	171.2 (7)	C10—C9—C8	121.89 (14)
F4—P1—F1A	65.9 (5)	C9^i^—C9—C8	118.85 (9)
F3—P1—F1A	154.6 (5)	C9—C10—C11	120.70 (15)
F6—P1—F1A	97.8 (4)	C9—C10—H10A	119.6
F2—P1—F1A	114.0 (5)	C11—C10—H10A	119.7
F5—P1—F1A	82.2 (4)	C11^i^—C11—C10	119.87 (10)
F2A—P1—F4A	175.5 (8)	C11^i^—C11—H11A	120.1
F3A—P1—F4A	86.9 (7)	C10—C11—H11A	120.1
F3—P1—F4A	70.3 (5)	N1—C12—C13	112.13 (14)
F1—P1—F4A	110.2 (5)	N1—C12—H12A	109.2
F6—P1—F4A	95.2 (5)	C13—C12—H12A	109.2
F2—P1—F4A	159.0 (6)	N1—C12—H12B	109.2
F5—P1—F4A	84.6 (5)	C13—C12—H12B	109.2
F1A—P1—F4A	85.1 (6)	H12A—C12—H12B	107.9
C1—N1—C2	108.21 (13)	C12—C13—H13A	109.5
C1—N1—C12	127.07 (14)	C12—C13—H13B	109.5
C2—N1—C12	124.68 (14)	H13A—C13—H13B	109.5
C1—N2—C7	108.18 (14)	C12—C13—H13C	109.5
C1—N2—C8	125.72 (14)	H13A—C13—H13C	109.5
C7—N2—C8	125.88 (14)	H13B—C13—H13C	109.5
N1—C1—N2	110.39 (14)		
			
C2—N1—C1—N2	−0.89 (19)	N1—C2—C7—C6	−178.67 (15)
C12—N1—C1—N2	−178.83 (15)	C3—C2—C7—N2	177.24 (15)
C7—N2—C1—N1	0.45 (19)	N1—C2—C7—N2	−0.69 (18)
C8—N2—C1—N1	175.33 (15)	C1—N2—C7—C6	177.89 (18)
C1—N1—C2—C7	0.97 (18)	C8—N2—C7—C6	3.0 (3)
C12—N1—C2—C7	178.98 (15)	C1—N2—C7—C2	0.18 (18)
C1—N1—C2—C3	−176.69 (18)	C8—N2—C7—C2	−174.70 (15)
C12—N1—C2—C3	1.3 (3)	C1—N2—C8—C9	−109.15 (18)
C7—C2—C3—C4	−0.5 (2)	C7—N2—C8—C9	64.8 (2)
N1—C2—C3—C4	176.88 (17)	N2—C8—C9—C10	9.8 (2)
C2—C3—C4—C5	1.2 (3)	N2—C8—C9—C9^i^	−169.32 (17)
C3—C4—C5—C6	−0.8 (3)	C9^i^—C9—C10—C11	3.6 (3)
C4—C5—C6—C7	−0.4 (3)	C8—C9—C10—C11	−175.57 (16)
C5—C6—C7—C2	1.2 (3)	C9—C10—C11—C11^i^	1.8 (3)
C5—C6—C7—N2	−176.25 (17)	C1—N1—C12—C13	15.8 (2)
C3—C2—C7—C6	−0.7 (3)	C2—N1—C12—C13	−161.87 (15)

Symmetry code: (i) −*x*, *y*, −*z*+3/2.

### Hydrogen-bond geometry (Å, º)

**Table:**

*D*—H···*A*	*D*—H	H···*A*	*D*···*A*	*D*—H···*A*
C1—H1*A*···F6^ii^	0.95	2.42	3.142 (3)	133
C3—H3*A*···F5^iii^	0.95	2.45	3.374 (2)	166
C5—H5*A*···F5^iv^	0.95	2.52	3.420 (3)	159
C6—H6*A*···F4^v^	0.95	2.53	3.392 (2)	151
C8—H8*B*···F1^ii^	0.99	2.39	3.350 (3)	164
C13—H13*C*···F1^vi^	0.98	2.55	3.523 (2)	174
C13—H13*C*···F5^vi^	0.98	2.50	3.166 (2)	125

Symmetry codes: (ii) *x*−1, *y*, *z*; (iii) *x*−1/2, −*y*+3/2, *z*+1/2; (iv) *x*−1/2, *y*−1/2, *z*; (v) −*x*+1, *y*, −*z*+3/2; (vi) −*x*+1, −*y*+2, −*z*+2.
